# Supplementary material for: HIVprotI: an integrated web based platform for prediction and design of HIV proteins inhibitors
Source: J Cheminform. 2018 Mar 9;10:12. doi: 10.1186/s13321-018-0266-y (PMC5845081; doi:10.1186/s13321-018-0266-y)
Supplement: Supplementary file 2 — Additional file 2. Source code of HIVProtI web server. [file 13321_2018_266_MOESM2_ESM.zip › HIVprotI_Source-code/hivprotI/weka-3-6-6/doc/weka/core/Instance.html]

Instance


---


|  |  |  |
| --- | --- | --- |
| **Overview** \| **Package** \| **Class** \| **Tree** \| **Deprecated** \| **Index** \| **Help** \| **Weka's home** | |  |
| **PREV CLASS**   **NEXT CLASS** | **FRAMES**    **NO FRAMES**     **All Classes** |  |
| SUMMARY: NESTED \| FIELD \| CONSTR \| METHOD | DETAIL: FIELD \| CONSTR \| METHOD |  |


---


## weka.core Class Instance

```
java.lang.Object
  weka.core.Instance
```

**All Implemented Interfaces:**: java.io.Serializable, Copyable, RevisionHandler

**Direct Known Subclasses:**: IndividualInstance, SparseInstance

---

``` public class Instance extends java.lang.Object implements Copyable, java.io.Serializable, RevisionHandler ```

Class for handling an instance. All values (numeric, date, nominal, string
or relational) are internally stored as floating-point numbers. If an
attribute is nominal (or a string or relational), the stored value is the
index of the corresponding nominal (or string or relational) value in the
attribute's definition. We have chosen this approach in favor of a more
elegant object-oriented approach because it is much faster.

Typical usage (code from the main() method of this class):

`...   
// Create empty instance with three attribute values   
Instance inst = new Instance(3);   
  
// Set instance's values for the attributes "length", "weight", and "position"  
inst.setValue(length, 5.3);   
inst.setValue(weight, 300);   
inst.setValue(position, "first");   
  
// Set instance's dataset to be the dataset "race"   
inst.setDataset(race);   
  
// Print the instance   
System.out.println("The instance: " + inst);   
...` 

All methods that change an instance are safe, ie. a change of an
instance does not affect any other instances. All methods that
change an instance's attribute values clone the attribute value
vector before it is changed. If your application heavily modifies
instance values, it may be faster to create a new instance from scratch.

**Version:**
:   $Revision: 5970 $

**Author:**
:   Eibe Frank (eibe@cs.waikato.ac.nz)

**See Also:**: Serialized Form

---

**Constructor Summary**

`Instance(double weight,
double[] attValues)`
  
          Constructor that inititalizes instance variable with given
values.

`Instance(Instance instance)`
  
          Constructor that copies the attribute values and the weight from
the given instance.

`Instance(int numAttributes)`
  
          Constructor of an instance that sets weight to one, all values to
be missing, and the reference to the dataset to null.


| **Method Summary** | |
| --- | --- |
| `Attribute` | `attribute(int index)`             Returns the attribute with the given index. |
| `Attribute` | `attributeSparse(int indexOfIndex)`             Returns the attribute with the given index. |
| `Attribute` | `classAttribute()`             Returns class attribute. |
| `int` | `classIndex()`             Returns the class attribute's index. |
| `boolean` | `classIsMissing()`             Tests if an instance's class is missing. |
| `double` | `classValue()`             Returns an instance's class value in internal format. |
| `java.lang.Object` | `copy()`             Produces a shallow copy of this instance. |
| `Instances` | `dataset()`             Returns the dataset this instance has access to. |
| `void` | `deleteAttributeAt(int position)`             Deletes an attribute at the given position (0 to numAttributes() - 1). |
| `java.util.Enumeration` | `enumerateAttributes()`             Returns an enumeration of all the attributes. |
| `boolean` | `equalHeaders(Instance inst)`             Tests if the headers of two instances are equivalent. |
| `java.lang.String` | `getRevision()`             Returns the revision string. |
| `boolean` | `hasMissingValue()`             Tests whether an instance has a missing value. |
| `int` | `index(int position)`             Returns the index of the attribute stored at the given position. |
| `void` | `insertAttributeAt(int position)`             Inserts an attribute at the given position (0 to numAttributes()). |
| `boolean` | `isMissing(Attribute att)`             Tests if a specific value is "missing". |
| `boolean` | `isMissing(int attIndex)`             Tests if a specific value is "missing". |
| `boolean` | `isMissingSparse(int indexOfIndex)`             Tests if a specific value is "missing". |
| `static boolean` | `isMissingValue(double val)`             Tests if the given value codes "missing". |
| `static void` | `main(java.lang.String[] options)`             Main method for testing this class. |
| `Instance` | `mergeInstance(Instance inst)`             Merges this instance with the given instance and returns the result. |
| `static double` | `missingValue()`             Returns the double that codes "missing". |
| `int` | `numAttributes()`             Returns the number of attributes. |
| `int` | `numClasses()`             Returns the number of class labels. |
| `int` | `numValues()`             Returns the number of values present. |
| `Instances` | `relationalValue(Attribute att)`             Returns the relational value of a relational attribute. |
| `Instances` | `relationalValue(int attIndex)`             Returns the relational value of a relational attribute. |
| `void` | `replaceMissingValues(double[] array)`             Replaces all missing values in the instance with the values contained in the given array. |
| `void` | `setClassMissing()`             Sets the class value of an instance to be "missing". |
| `void` | `setClassValue(double value)`             Sets the class value of an instance to the given value (internal floating-point format). |
| `void` | `setClassValue(java.lang.String value)`             Sets the class value of an instance to the given value. |
| `void` | `setDataset(Instances instances)`             Sets the reference to the dataset. |
| `void` | `setMissing(Attribute att)`             Sets a specific value to be "missing". |
| `void` | `setMissing(int attIndex)`             Sets a specific value to be "missing". |
| `void` | `setValue(Attribute att, double value)`             Sets a specific value in the instance to the given value (internal floating-point format). |
| `void` | `setValue(Attribute att, java.lang.String value)`             Sets a value of an nominal or string attribute to the given value. |
| `void` | `setValue(int attIndex, double value)`             Sets a specific value in the instance to the given value (internal floating-point format). |
| `void` | `setValue(int attIndex, java.lang.String value)`             Sets a value of a nominal or string attribute to the given value. |
| `void` | `setValueSparse(int indexOfIndex, double value)`             Sets a specific value in the instance to the given value (internal floating-point format). |
| `void` | `setWeight(double weight)`             Sets the weight of an instance. |
| `java.lang.String` | `stringValue(Attribute att)`             Returns the value of a nominal, string, date, or relational attribute for the instance as a string. |
| `java.lang.String` | `stringValue(int attIndex)`             Returns the value of a nominal, string, date, or relational attribute for the instance as a string. |
| `double[]` | `toDoubleArray()`             Returns the values of each attribute as an array of doubles. |
| `java.lang.String` | `toString()`             Returns the description of one instance. |
| `java.lang.String` | `toString(Attribute att)`             Returns the description of one value of the instance as a string. |
| `java.lang.String` | `toString(int attIndex)`             Returns the description of one value of the instance as a string. |
| `double` | `value(Attribute att)`             Returns an instance's attribute value in internal format. |
| `double` | `value(int attIndex)`             Returns an instance's attribute value in internal format. |
| `double` | `valueSparse(int indexOfIndex)`             Returns an instance's attribute value in internal format. |
| `double` | `weight()`             Returns the instance's weight. |

**Methods inherited from class java.lang.Object**

`equals, getClass, hashCode, notify, notifyAll, wait, wait, wait`

**Constructor Detail**

### Instance

```
public Instance(Instance instance)
```

:   Constructor that copies the attribute values and the weight from
    the given instance. Reference to the dataset is set to null.
    (ie. the instance doesn't have access to information about the
    attribute types)

    **Parameters:**: `instance` - the instance from which the attribute values and the weight are to be copied

---


### Instance

```
public Instance(double weight,
                double[] attValues)
```

:   Constructor that inititalizes instance variable with given
    values. Reference to the dataset is set to null. (ie. the instance
    doesn't have access to information about the attribute types)

    **Parameters:**: `weight` - the instance's weight: `attValues` - a vector of attribute values

---


### Instance

```
public Instance(int numAttributes)
```

:   Constructor of an instance that sets weight to one, all values to
    be missing, and the reference to the dataset to null. (ie. the instance
    doesn't have access to information about the attribute types)

    **Parameters:**: `numAttributes` - the size of the instance


**Method Detail**

### attribute

```
public Attribute attribute(int index)
```

:   Returns the attribute with the given index.

    :   **Parameters:**: `index` - the attribute's index **Returns:**: the attribute at the given position **Throws:**: `UnassignedDatasetException` - if instance doesn't have access to a dataset

---


### attributeSparse

```
public Attribute attributeSparse(int indexOfIndex)
```

:   Returns the attribute with the given index. Does the same
    thing as attribute().

    :   **Parameters:**: `indexOfIndex` - the index of the attribute's index **Returns:**: the attribute at the given position **Throws:**: `UnassignedDatasetException` - if instance doesn't have access to a dataset

---


### classAttribute

```
public Attribute classAttribute()
```

:   Returns class attribute.

    :   **Returns:**: the class attribute **Throws:**: `UnassignedDatasetException` - if the class is not set or the instance doesn't have access to a dataset

---


### classIndex

```
public int classIndex()
```

:   Returns the class attribute's index.

    :   **Returns:**: the class index as an integer **Throws:**: `UnassignedDatasetException` - if instance doesn't have access to a dataset

---


### classIsMissing

```
public boolean classIsMissing()
```

:   Tests if an instance's class is missing.

    :   **Returns:**: true if the instance's class is missing **Throws:**: `UnassignedClassException` - if the class is not set or the instance doesn't have access to a dataset

---


### classValue

```
public double classValue()
```

:   Returns an instance's class value in internal format. (ie. as a
    floating-point number)

    :   **Returns:**: the corresponding value as a double (If the corresponding attribute is nominal (or a string) then it returns the value's index as a double). **Throws:**: `UnassignedClassException` - if the class is not set or the instance doesn't have access to a dataset

---


### copy

```
public java.lang.Object copy()
```

:   Produces a shallow copy of this instance. The copy has
    access to the same dataset. (if you want to make a copy
    that doesn't have access to the dataset, use
    `new Instance(instance)`

    :   **Specified by:**: `copy` in interface `Copyable`
    :   **Returns:**: the shallow copy

---


### dataset

```
public Instances dataset()
```

:   Returns the dataset this instance has access to. (ie. obtains
    information about attribute types from) Null if the instance
    doesn't have access to a dataset.

    :   **Returns:**: the dataset the instance has accesss to

---


### deleteAttributeAt

```
public void deleteAttributeAt(int position)
```

:   Deletes an attribute at the given position (0 to
    numAttributes() - 1). Only succeeds if the instance does not
    have access to any dataset because otherwise inconsistencies
    could be introduced.

    :   **Parameters:**: `position` - the attribute's position **Throws:**: `java.lang.RuntimeException` - if the instance has access to a dataset

---


### enumerateAttributes

```
public java.util.Enumeration enumerateAttributes()
```

:   Returns an enumeration of all the attributes.

    :   **Returns:**: enumeration of all the attributes **Throws:**: `UnassignedDatasetException` - if the instance doesn't have access to a dataset

---


### equalHeaders

```
public boolean equalHeaders(Instance inst)
```

:   Tests if the headers of two instances are equivalent.

    :   **Parameters:**: `inst` - another instance **Returns:**: true if the header of the given instance is equivalent to this instance's header **Throws:**: `UnassignedDatasetException` - if instance doesn't have access to any dataset

---


### hasMissingValue

```
public boolean hasMissingValue()
```

:   Tests whether an instance has a missing value. Skips the class attribute if set.

    :   **Returns:**: true if instance has a missing value. **Throws:**: `UnassignedDatasetException` - if instance doesn't have access to any dataset

---


### index

```
public int index(int position)
```

:   Returns the index of the attribute stored at the given position.
    Just returns the given value.

    :   **Parameters:**: `position` - the position **Returns:**: the index of the attribute stored at the given position

---


### insertAttributeAt

```
public void insertAttributeAt(int position)
```

:   Inserts an attribute at the given position (0 to
    numAttributes()). Only succeeds if the instance does not
    have access to any dataset because otherwise inconsistencies
    could be introduced.

    :   **Parameters:**: `position` - the attribute's position **Throws:**: `java.lang.RuntimeException` - if the instance has accesss to a dataset: `java.lang.IllegalArgumentException` - if the position is out of range

---


### isMissing

```
public boolean isMissing(int attIndex)
```

:   Tests if a specific value is "missing".

    :   **Parameters:**: `attIndex` - the attribute's index **Returns:**: true if the value is "missing"

---


### isMissingSparse

```
public boolean isMissingSparse(int indexOfIndex)
```

:   Tests if a specific value is "missing". Does
    the same thing as isMissing() if applied to an Instance.

    :   **Parameters:**: `indexOfIndex` - the index of the attribute's index **Returns:**: true if the value is "missing"

---


### isMissing

```
public boolean isMissing(Attribute att)
```

:   Tests if a specific value is "missing".
    The given attribute has to belong to a dataset.

    :   **Parameters:**: `att` - the attribute **Returns:**: true if the value is "missing"

---


### isMissingValue

```
public static boolean isMissingValue(double val)
```

:   Tests if the given value codes "missing".

    :   **Parameters:**: `val` - the value to be tested **Returns:**: true if val codes "missing"

---


### mergeInstance

```
public Instance mergeInstance(Instance inst)
```

:   Merges this instance with the given instance and returns
    the result. Dataset is set to null.

    :   **Parameters:**: `inst` - the instance to be merged with this one **Returns:**: the merged instances

---


### missingValue

```
public static double missingValue()
```

:   Returns the double that codes "missing".

    :   **Returns:**: the double that codes "missing"

---


### numAttributes

```
public int numAttributes()
```

:   Returns the number of attributes.

    :   **Returns:**: the number of attributes as an integer

---


### numClasses

```
public int numClasses()
```

:   Returns the number of class labels.

    :   **Returns:**: the number of class labels as an integer if the class attribute is nominal, 1 otherwise. **Throws:**: `UnassignedDatasetException` - if instance doesn't have access to any dataset

---


### numValues

```
public int numValues()
```

:   Returns the number of values present. Always the same as numAttributes().

    :   **Returns:**: the number of values

---


### replaceMissingValues

```
public void replaceMissingValues(double[] array)
```

:   Replaces all missing values in the instance with the
    values contained in the given array. A deep copy of
    the vector of attribute values is performed before the
    values are replaced.

    :   **Parameters:**: `array` - containing the means and modes **Throws:**: `java.lang.IllegalArgumentException` - if numbers of attributes are unequal

---


### setClassMissing

```
public void setClassMissing()
```

:   Sets the class value of an instance to be "missing". A deep copy of
    the vector of attribute values is performed before the
    value is set to be missing.

    :   **Throws:**: `UnassignedClassException` - if the class is not set: `UnassignedDatasetException` - if the instance doesn't have access to a dataset

---


### setClassValue

```
public void setClassValue(double value)
```

:   Sets the class value of an instance to the given value (internal
    floating-point format). A deep copy of the vector of attribute
    values is performed before the value is set.

    :   **Parameters:**: `value` - the new attribute value (If the corresponding attribute is nominal (or a string) then this is the new value's index as a double). **Throws:**: `UnassignedClassException` - if the class is not set: `UnaddignedDatasetException` - if the instance doesn't have access to a dataset

---


### setClassValue

```
public final void setClassValue(java.lang.String value)
```

:   Sets the class value of an instance to the given value. A deep
    copy of the vector of attribute values is performed before the
    value is set.

    :   **Parameters:**: `value` - the new class value (If the class is a string attribute and the value can't be found, the value is added to the attribute). **Throws:**: `UnassignedClassException` - if the class is not set: `UnassignedDatasetException` - if the dataset is not set: `java.lang.IllegalArgumentException` - if the attribute is not nominal or a string, or the value couldn't be found for a nominal attribute

---


### setDataset

```
public final void setDataset(Instances instances)
```

:   Sets the reference to the dataset. Does not check if the instance
    is compatible with the dataset. Note: the dataset does not know
    about this instance. If the structure of the dataset's header
    gets changed, this instance will not be adjusted automatically.

    :   **Parameters:**: `instances` - the reference to the dataset

---


### setMissing

```
public final void setMissing(int attIndex)
```

:   Sets a specific value to be "missing". Performs a deep copy
    of the vector of attribute values before the value is set to
    be missing.

    :   **Parameters:**: `attIndex` - the attribute's index

---


### setMissing

```
public final void setMissing(Attribute att)
```

:   Sets a specific value to be "missing". Performs a deep copy
    of the vector of attribute values before the value is set to
    be missing. The given attribute has to belong to a dataset.

    :   **Parameters:**: `att` - the attribute

---


### setValue

```
public void setValue(int attIndex,
                     double value)
```

:   Sets a specific value in the instance to the given value
    (internal floating-point format). Performs a deep copy
    of the vector of attribute values before the value is set.

    :   **Parameters:**: `attIndex` - the attribute's index: `value` - the new attribute value (If the corresponding attribute is nominal (or a string) then this is the new value's index as a double).

---


### setValueSparse

```
public void setValueSparse(int indexOfIndex,
                           double value)
```

:   Sets a specific value in the instance to the given value
    (internal floating-point format). Performs a deep copy
    of the vector of attribute values before the value is set.
    Does exactly the same thing as setValue().

    :   **Parameters:**: `indexOfIndex` - the index of the attribute's index: `value` - the new attribute value (If the corresponding attribute is nominal (or a string) then this is the new value's index as a double).

---


### setValue

```
public final void setValue(int attIndex,
                           java.lang.String value)
```

:   Sets a value of a nominal or string attribute to the given
    value. Performs a deep copy of the vector of attribute values
    before the value is set.

    :   **Parameters:**: `attIndex` - the attribute's index: `value` - the new attribute value (If the attribute is a string attribute and the value can't be found, the value is added to the attribute). **Throws:**: `UnassignedDatasetException` - if the dataset is not set: `java.lang.IllegalArgumentException` - if the selected attribute is not nominal or a string, or the supplied value couldn't be found for a nominal attribute

---


### setValue

```
public final void setValue(Attribute att,
                           double value)
```

:   Sets a specific value in the instance to the given value
    (internal floating-point format). Performs a deep copy of the
    vector of attribute values before the value is set, so if you are
    planning on calling setValue many times it may be faster to
    create a new instance using toDoubleArray. The given attribute
    has to belong to a dataset.

    :   **Parameters:**: `att` - the attribute: `value` - the new attribute value (If the corresponding attribute is nominal (or a string) then this is the new value's index as a double).

---


### setValue

```
public final void setValue(Attribute att,
                           java.lang.String value)
```

:   Sets a value of an nominal or string attribute to the given
    value. Performs a deep copy of the vector of attribute values
    before the value is set, so if you are planning on calling setValue many
    times it may be faster to create a new instance using toDoubleArray.
    The given attribute has to belong to a dataset.

    :   **Parameters:**: `att` - the attribute: `value` - the new attribute value (If the attribute is a string attribute and the value can't be found, the value is added to the attribute). **Throws:**: `java.lang.IllegalArgumentException` - if the the attribute is not nominal or a string, or the value couldn't be found for a nominal attribute

---


### setWeight

```
public final void setWeight(double weight)
```

:   Sets the weight of an instance.

    :   **Parameters:**: `weight` - the weight

---


### relationalValue

```
public final Instances relationalValue(int attIndex)
```

:   Returns the relational value of a relational attribute.

    :   **Parameters:**: `attIndex` - the attribute's index **Returns:**: the corresponding relation as an Instances object **Throws:**: `java.lang.IllegalArgumentException` - if the attribute is not a relation-valued attribute: `UnassignedDatasetException` - if the instance doesn't belong to a dataset.

---


### relationalValue

```
public final Instances relationalValue(Attribute att)
```

:   Returns the relational value of a relational attribute.

    :   **Parameters:**: `att` - the attribute **Returns:**: the corresponding relation as an Instances object **Throws:**: `java.lang.IllegalArgumentException` - if the attribute is not a relation-valued attribute: `UnassignedDatasetException` - if the instance doesn't belong to a dataset.

---


### stringValue

```
public final java.lang.String stringValue(int attIndex)
```

:   Returns the value of a nominal, string, date, or relational attribute
    for the instance as a string.

    :   **Parameters:**: `attIndex` - the attribute's index **Returns:**: the value as a string **Throws:**: `java.lang.IllegalArgumentException` - if the attribute is not a nominal, string, date, or relation-valued attribute.: `UnassignedDatasetException` - if the instance doesn't belong to a dataset.

---


### stringValue

```
public final java.lang.String stringValue(Attribute att)
```

:   Returns the value of a nominal, string, date, or relational attribute
    for the instance as a string.

    :   **Parameters:**: `att` - the attribute **Returns:**: the value as a string **Throws:**: `java.lang.IllegalArgumentException` - if the attribute is not a nominal, string, date, or relation-valued attribute.: `UnassignedDatasetException` - if the instance doesn't belong to a dataset.

---


### toDoubleArray

```
public double[] toDoubleArray()
```

:   Returns the values of each attribute as an array of doubles.

    :   **Returns:**: an array containing all the instance attribute values

---


### toString

```
public java.lang.String toString()
```

:   Returns the description of one instance. If the instance
    doesn't have access to a dataset, it returns the internal
    floating-point values. Quotes string
    values that contain whitespace characters.

    :   **Overrides:**: `toString` in class `java.lang.Object`
    :   **Returns:**: the instance's description as a string

---


### toString

```
public final java.lang.String toString(int attIndex)
```

:   Returns the description of one value of the instance as a
    string. If the instance doesn't have access to a dataset, it
    returns the internal floating-point value. Quotes string
    values that contain whitespace characters, or if they
    are a question mark.

    :   **Parameters:**: `attIndex` - the attribute's index **Returns:**: the value's description as a string

---


### toString

```
public final java.lang.String toString(Attribute att)
```

:   Returns the description of one value of the instance as a
    string. If the instance doesn't have access to a dataset it
    returns the internal floating-point value. Quotes string
    values that contain whitespace characters, or if they
    are a question mark.
    The given attribute has to belong to a dataset.

    :   **Parameters:**: `att` - the attribute **Returns:**: the value's description as a string

---


### value

```
public double value(int attIndex)
```

:   Returns an instance's attribute value in internal format.

    :   **Parameters:**: `attIndex` - the attribute's index **Returns:**: the specified value as a double (If the corresponding attribute is nominal (or a string) then it returns the value's index as a double).

---


### valueSparse

```
public double valueSparse(int indexOfIndex)
```

:   Returns an instance's attribute value in internal format.
    Does exactly the same thing as value() if applied to an Instance.

    :   **Parameters:**: `indexOfIndex` - the index of the attribute's index **Returns:**: the specified value as a double (If the corresponding attribute is nominal (or a string) then it returns the value's index as a double).

---


### value

```
public double value(Attribute att)
```

:   Returns an instance's attribute value in internal format.
    The given attribute has to belong to a dataset.

    :   **Parameters:**: `att` - the attribute **Returns:**: the specified value as a double (If the corresponding attribute is nominal (or a string) then it returns the value's index as a double).

---


### weight

```
public final double weight()
```

:   Returns the instance's weight.

    :   **Returns:**: the instance's weight as a double

---


### main

```
public static void main(java.lang.String[] options)
```

:   Main method for testing this class.

    :   **Parameters:**: `options` - the commandline options - ignored

---


### getRevision

```
public java.lang.String getRevision()
```

:   Returns the revision string.

    :   **Specified by:**: `getRevision` in interface `RevisionHandler`
    :   **Returns:**: the revision


---


|  |  |  |
| --- | --- | --- |
| **Overview** \| **Package** \| **Class** \| **Tree** \| **Deprecated** \| **Index** \| **Help** \| **Weka's home** | |  |
| **PREV CLASS**   **NEXT CLASS** | **FRAMES**    **NO FRAMES**     **All Classes** |  |
| SUMMARY: NESTED \| FIELD \| CONSTR \| METHOD | DETAIL: FIELD \| CONSTR \| METHOD |  |


---
